# Differentiation of respiratory epithelium in a 3-dimensional co-culture with fibroblasts embedded in fibrin gel

**DOI:** 10.1186/s40248-016-0046-3

**Published:** 2016-03-01

**Authors:** Stefanie Albers, Anja Lena Thiebes, Kai L. Gessenich, Stefan Jockenhoevel, Christian G. Cornelissen

**Affiliations:** 1 Department for Internal Medicine – Section for Pneumology, University Hospital Aachen, Pauwelsstraße 30, Aachen, Germany; 2 Department of Tissue Engineering & Textile Implants, Institute for Applied Medical Engineering, Helmholtz Institute of the RWTH University Hospital, Pauwelsstr. 20, 52074 Aachen, Germany

**Keywords:** Airways, Fibrin gel, Fibroblast, Respiratory epithelium, Tissue engineering

## Abstract

**Background:**

Tracheal tissue engineering is a promising option for the treatment of tracheal defects. In a previous study we proved the suitability of fibrin gel as a scaffold for tracheal tissue engineering. This study investigates whether the differentiation of respiratory epithelium can be increased by culturing epithelial cells in a three dimensional system containing fibroblasts embedded into fibrin gel.

**Methods:**

Respiratory epithelial cells were isolated from porcine trachea, seeded onto a fibrin gel and kept in air-liquid-interface culture for 33 days. Morphology as well as pan-cytokeratin, MUC5AC and claudin-1 expression of cells cultured on pure fibrin gel were compared to culture on gels containing fibroblasts.

**Results:**

After two weeks, cells seeded on pure fibrin gel were multilayered, showed hyperproliferation and dedifferentiation. Co-cultured cells built up a pseudostratified epithelium. The differentiation and organization of epithelial structure improved with respect to time. After four weeks, morphology of the co-cultured respiratory epithelium resembled native tracheal epithelium. Immunohistochemistry showed that respiratory epithelium co-cultured with fibroblasts had an increasing similarity of pan-cytokeratin expression compared to native trachea. Cells cultured without fibroblasts differed in pan-cytokeratin expression from native trachea and did not show any improvement of differentiation. Immunohistochemical staining of MUC5AC and claudin-1 proved seeded cells being respiratory epithelial cells.

**Conclusions:**

This study indicates that adding fibroblasts to fibrin gel positively influences the differentiation of respiratory epithelium.

## Background

Long segment tracheomalacia and tracheostenosis are diseases with significant morbidity and a high mortality rate, since they affect respiration and mucociliary function of the trachea [1]. They mandate surgical treatment or stent implantation to ensure an adequate quality of life or even to permit survival of the patient. Long segment tracheal reconstruction requires replacement tissues, for example costal cartilage, [2] since an end to end anastomosis can create tension on the trachea. Harvesting costal cartilage might cause donor site morbidity and necessitates several surgical procedures.

Tracheal tissue engineering is a promising option for the treatment of tracheal defects. A tissue engineered construct might conserve mucociliary function and could be a main advantage for the patient. It would help to overcome the shortage of donor organs in allogenic transplantation and to prevent the patient's lifelong immunosuppressive treatment. Macchiarini et al. successfully implanted a decellularized tracheal homograft seeded with respiratory epithelial cells [3]. Since decellularized matrices also need donor organs this approach might compete with organs required for transplantation. In their last proof of concept study, this group transplanted a stem-cell-seeded bioartificial nanocomposite into a 36 year old tracheal cancer patient [4].

Scaffolds in tracheal tissue engineering can also be produced from natural polymers such as collagen or fibrin gel. We already demonstrated the suitability of fibrin gel as a scaffold for tracheal tissue engineering [5]. Proliferation, functionality and differentiation of respiratory epithelial cells grown on fibrin gel were compared to cells cultured on a collagen-coated, microporous membrane. The study revealed no significant differences in differentiation, functionality or proliferation. We concluded that fibrin gel can be an approach in developing a scaffold for tracheal tissue engineering, as it can be produced autologously. Injection molding can be used to create complex geometries and its degradation can be controlled by protease inhibitors [6].

Two dimensional co-culture systems with fibroblasts are an established method to improve the differentiation of respiratory epithelium. Goto et al. co-cultured fibroblasts and respiratory epithelial cells in an air-liquid-interface (ALI) culture system, where both cell types were seeded onto different sites of an amnion membrane. They found positive effects on morphology of the cells [7]. Also, it is a common culture system to investigate molecular mechanisms of asthma [8].

We hypothesized that co-culture with fibroblasts might improve the differentiation of respiratory epithelium in a three-dimensional tissue model based on fibroblasts embedded into fibrin gel.

## Methods

### Experimental setup

Respiratory epithelial cells were seeded either onto pure fibrin gels or onto fibrin gels containing fibroblasts. The constructs were cultured on membrane inserts for up to 33 days, assessing cell proliferation and differentiation after 7, 14, 21 and 33 days. ALI culture conditions were established as soon as transepithelial electrical resistance suggested full epithelial coverage of the constructs. All experiments were performed in triplicate for every point of time of analysis.

### Isolation and expansion of respiratory epithelial cells

Parts of porcine trachea were harvested from pigs euthanized for other purposes at the animal facilities in the University Hospital Aachen. Cell harvesting was approved by the local ethical committee. Cells were isolated according to a protocol first published by Widdicombe et al [9]. The trachea was split longitudinally cutting through the pars membranacea and opposite to the first cut. Subsequently the mucosa was incised longitudinally. The mucosa stripes were removed and placed into a solution of protease XIV (Sigma, Germany) at 0.4 mg/mL. The stripes were incubated at 4 °C over night. After removal of the stripes and centrifugation (200 g, 5 min) the cells were dispersed in Dulbecco’s Modified Essential Medium (DMEM, Sigma, Germany) containing 10 % fetal calf serum (FCS, PAA, Austria) and plated at 2*10^4^ cells/cm^2^. After 24 h, medium was changed to Gray’s medium, which is a 1:1 mixture of DMEM and LHC-9 (Invitrogen, USA). 1.5 μg/mL of bovine serum albumin were added (Sigma, Germany). When cells reached 70 % of confluence, cells were passaged using 0.5 mg/mL Trypsin/0.22 mg/mL EDTA solution (PAA, Austria) for detachment. Cells from the second passage were used for the experiment.

### Isolation and expansion of fibroblasts

Remaining parts of the trachea were cut into small pieces and placed into Trypsin/EDTA for 15 min at 37 °C. After stopping the enzymatic reaction by addition of DMEM containing 10 % FCS, pieces were removed and cells were centrifuged at 500 g for 5 min. Cells were dispersed in DMEM containing 10 % FCS and plated at a density of 2*10^4^ cells/cm^2^. Medium was exchanged every 72 h and cells were passaged when they reached 70 % of confluence. Cells from passage 4 were used for the experiment.

### Fabrication of fibrin gel and embedding of fibroblasts

Lypophilized human fibrin gel (plasminogen free; Sigma, Germany) was dissolved in purified water at a concentration of 25 mg/mL and dialyzed using a dialysis membrane (Novodirect, Germany) with a cut-off of 6000–8000 MW overnight against Trizma buffered saline (TBS). This contains 4.91 g/L Trizma HCL, 0.72 g/L Trizma Base, 9.00 g/L NaCl and 0.23 g/L KCl in double-distilled water at a pH of 7.4 (all from Sigma, Germany). A spectrophotometer (Tecan infinity reader, Tecan, Switzerland) was used to measure absorbance at 280 nm to examine the fibrinogen concentration after sterile filtration. For polymerization of the fibrin gel, a polymerization solution was composed, containing TBS, calcium chloride (50 mmol/L) and thrombin (40 U/mL). For embedding fibroblasts, cells from passage 4 were trypsinized. After centrifugation, they were resuspended in TBS and added to the polymerization solution with a final concentration of 10*10^6^ cells/mL fibrin gel. Transwell™ inserts (24-well, pore size 0.2 μm, polyethylene; Corning, USA) were covered with 125 μL fibrin gel using a 1:1 mixture of polymerization solution and fibrinogen solution. Thus, the final concentration of fibrinogen in the fibrin gel was 12.5 mg/mL. 1.6 μL of the protease inhibitor tranexamic acid (Bayer, Germany) were added per mL of gel to inhibit fibrin gel degradation. The gels were incubated at room temperature for 45 min to allow for polymerization of the fibrin network.

### Respiratory epithelial cell seeding and subsequent culture of the constructs

Cells from the second passage were harvested and dispersed in Gray’s medium at 1.5*10^5^ cells per mL and seeded at a density of 8*10^4^ cells/cm^2^ onto pure fibrin gel or fibrin gel containing fibroblasts as described above. Cell culture medium was changed every 24 h. ALI conditions were employed after two days.

### Assessment of cell differentiation

Samples of cultured cells were taken after 7, 14, 21 and 33 days for histologic and immunohistochemical analysis. Samples and native porcine trachea were fixed in Carnoy’s fixative, embedded in paraffin and sectioned at 3 μm thickness. Sections were stained by standard periodic acid Schiff’s reaction (PAS) protocol and for analysis of general epithelial morphology. Sections were analyzed by routine bright field light microscopy. For immunohistochemical analysis, Carnoy’s fixed paraffin embedded sections were used. Nonspecific sites were blocked with 5 % normal goat serum (NGS; Sigma) and cells permeabilized in 0.1 % Triton-PBS. Then sections were incubated with the first antibody for 1 h at 37 °C. After washing steps, the sections were incubated with the secondary antibody for 1 h at room temperature and the nuclei were counterstained with DAPI (Molecular Probes). A solution of 1 g bovine serum albumin and 0.1 g sodium azide (Sigma, Germany) in 100 mL of phosphate buffered saline (PAA, Germany) was used as antibody diluent. For the secondary antibody diluent 2 % of normal goat serum (Millipore, Germany) was added to this solution. As primary antibodies we used rabbit anti-pan-cytokeratin (1:100, Acris, Germany), rabbit anti-claudin-1 (1:100, Biorbyt,United Kingdom) and mouse anti - MUC5AC (1, 400, Acris, Germany). For the negative controls, the samples were incubated with the respective IgG isotype control (Acris and ThermoFisher, Germany) with the same concentration as the first antibody. As secondary antibody AlexaFluor-488 goat anti-rabbit, AlexaFluor-594 goat anti-rabbit, AlexaFluor-594 goat anti-mouse (all Invitrogen, Germany) were used, respectively. The samples were analyzed using a microscope equipped for epi-illumination (Axio Imager; Carl Zeiss GmbH, Germany). To achieve comparable results the same exposure time was chosen for all samples.

### Quantification of cell differentiation

For a quantification of cell differentiation all cells were counted in 3 slices stained with PAS of each sample. We defined four groups of cell differentiation:

A. Similar to ciliated cells. Group A cells are defined as cells reaching the surface and not being in a flat shape.
B. Similar to basal cells. Group B cells are defined as cells touching fibrin gel and being in a round shape.
C. Strongly PAS positive cells.
D. Cells without affiliation. They are defined as cells that have no relation to surface or fibrin gel or flattened or do not belong to group A-C.

The percentages of cells occurring for each condition are compared.

### Statistical analysis

Statistical analysis was performed on the results of cell quantification. Continuous variables are expressed as mean ± standard deviation. Data analysis was performed using commercially available software (Microsoft Office Excel, The Microsoft Corporation, USA & SAS enterprise guide version 4, SAS Institute Inc., USA).

## Results

### PAS-reaction

Cells seeded onto fibrin gel without fibroblasts built up a monolayer of cells by day 7. On day 14, we observed multiple cell layers. On day 33 increasing numbers of cell layers were observed, resulting in hyperproliferation compared to native trachea. Morphologically, cells grown on pure fibrin gel did not show clear differences between cell types. Still, the apical cells appeared rather polygonal with a large cell body. Cells in the middle layer displayed a flat shape with smaller cell bodies. Cells located at the bottom of the epithelium were observed to be the smallest, scarce in cytoplasm.

Epithelial cells co-cultured with fibroblasts did not show hyperproliferation. Instead, after 14 days of cultivation cells were organized in pseudostratified cell layers, resembling the organization of epithelium in native trachea.

Cells grown on fibrin gel with embedded fibroblasts displayed clearly differentiable cell types. Basal cells were round, with a small nuclear-to-cytosol-ratio, columnar cells reached to the apical surface. Mucus-producing cells, which can be detected by PAS-staining, were found between ciliated cells. All cell types showed similarities to cells found in native trachea.

**Fig. 1:**
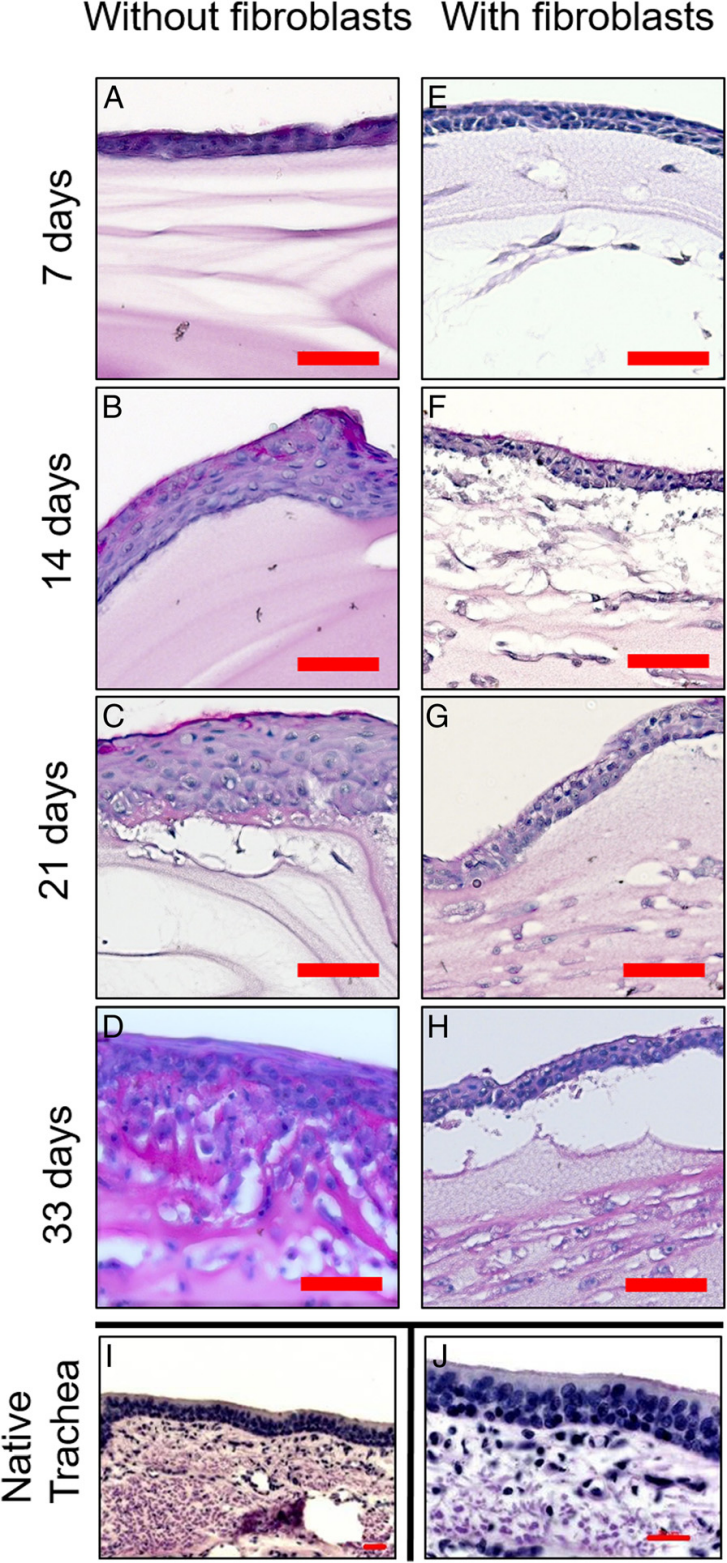
PAS staining. **a**-**d** PAS staining of respiratory epithelial cells seeded onto pure fibrin gel after 7, 14, 21, 33 days, respectively; Cells seeded onto fibrin gel without fibroblasts built up a monolayer of cells by day 7 (**a**). On day 14, we observed multiple cell layers (**b**). On day 33 (**d**) increasing numbers of cell layers were observed, resulting in hyperproliferation compared to native trachea (**i**). Morphologically, cells grown on pure fibrin gel did not show clear differences between cell types. Respiratory epithelial cells grown upon pure fibrin gel lose organization into pseudostratified epithelium over time, possibly due to dedifferentiation. **e**-**h** PAS staining of respiratory epithelial cells seeded onto fibrin gel with embedded fibroblasts after 7, 14, 21 and 33 days. Cells did not show hyperproliferation. Instead, after 14 days of cultivation cells were organized in pseudostratified cell layers (**f**), almost resembling the organization of epithelium in native trachea (**i**). A continuous surface of ciliated cells in samples with embedded fibroblasts persists until day 33 (**h**). **i, j** Different magnifications of PAS-staining of native porcine trachea, a pseudostratified epithelium with PAS-positive goblet cells, in the submucosa vasculature structures can be found. Scale: 50 μm

**Fig. 2:**
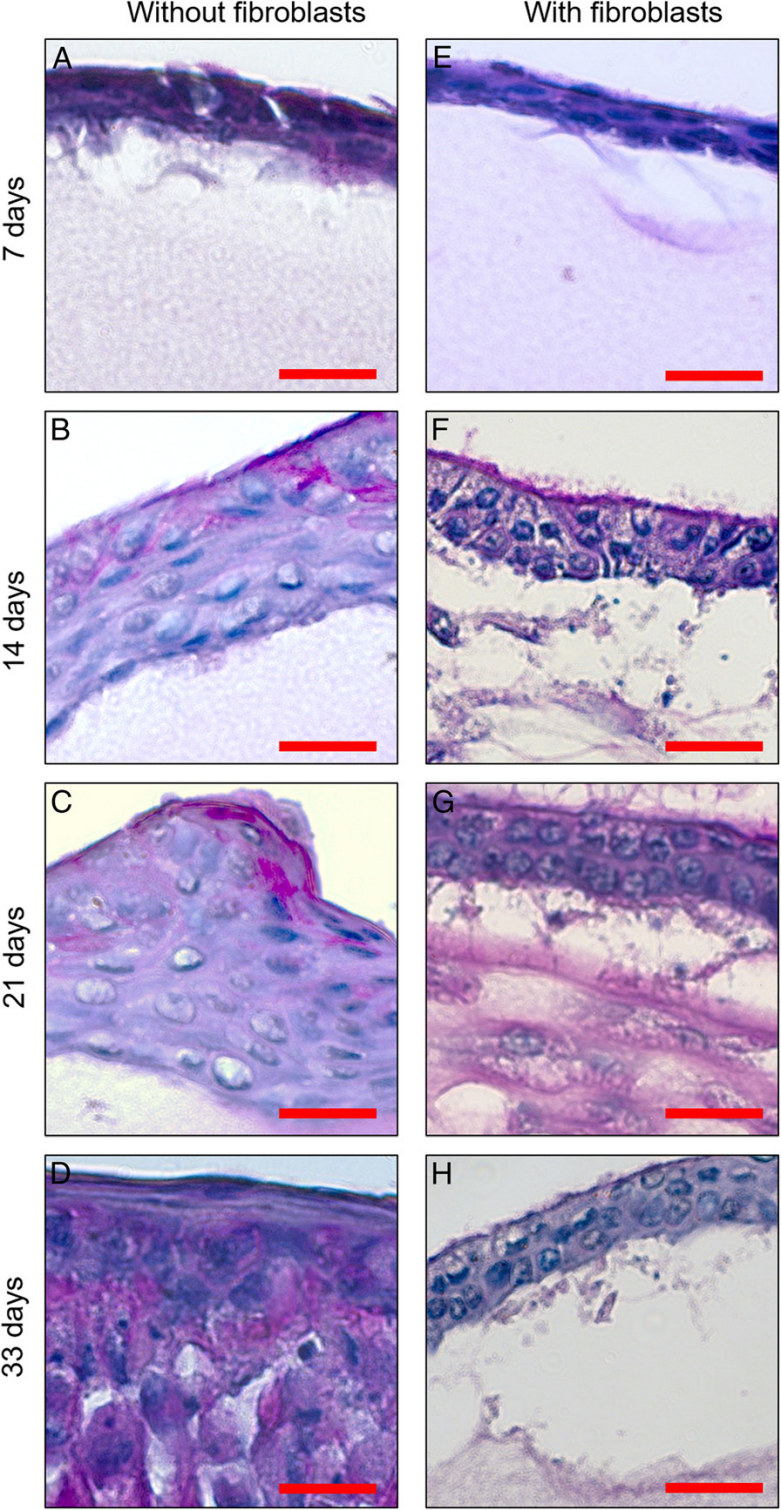
Higher magnification of PAS staining. **a**-**d** Detail of Fig. 1 in higher magnification of cells seeded onto pure fibrin gel on day 7 (**a**), 14 (**b**), 21 (**c**) and 33 (**d**). **e**-**h** Detail of Fig. 1 in higher magnification of respiratory epithelial cells grown on fibrin gel with embedded fibroblasts on day 7 (**e**), 14 (**f**), 21 (**g**) and 33 (**h**). Scale: 20 μm

Ciliated cells and goblet cells were observed from day 14 in both samples. A continuous surface of ciliated cells in samples with embedded fibroblasts persists until day 33. Respiratory epithelial cells grown upon pure fibrin gel lose cilia over time; possibly due to dedifferentiation (see Figs. 1 and 2).

### Quantification of cell differentiation

**Fig. 3:**
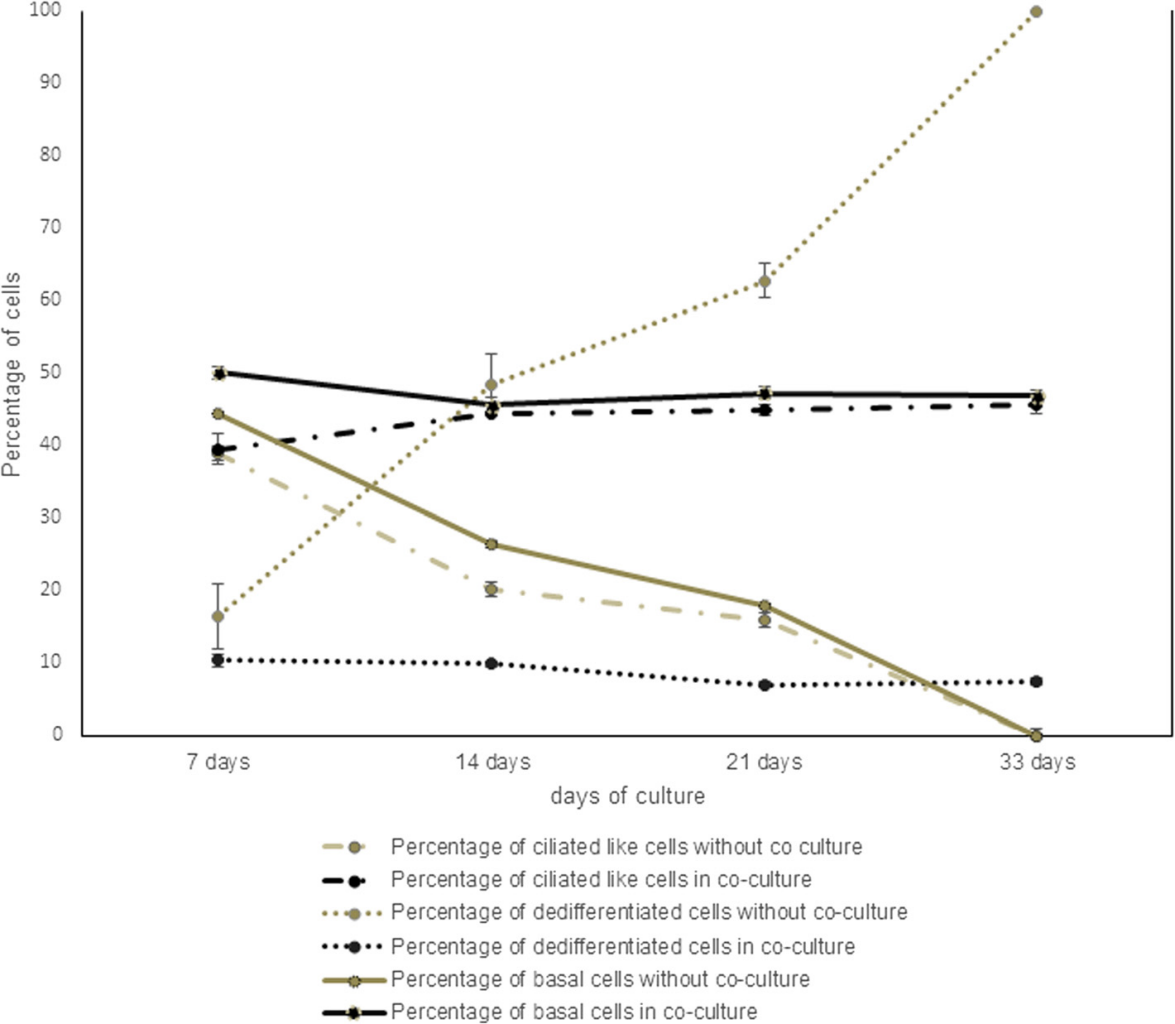
Graph of percentages of cell differentiation. Graph of percentages of cell differentiation of cells grown on pure fibrin gel and cells grown on fibrin gel with embedded fibroblasts. Cells grown on pure fibrin gel show a clear increase in dedifferentiated cells over time. Cells grown on fibrin gel with embedded fibroblast show a stable ratio of ciliated-like cells and dedifferentiated cells

Figure 3 shows an increase in dedifferentiated cells for respiratory epithelial cells grown on pure fibrin gel, as well as a decrease in the percentage of ciliated-like cells over time. After 33 days of culture all cells are dedifferentiated. Strongly PAS positive cells are found after 14 and 21 days of culture as seen in detail in Table 1.

**Table 1:** Quantification of cell differentiation between cells cultured without fibroblasts and cells in co-culture

Percentage of cells belonging to	7 days	l4 days	21 days	33 days
Group A				
Cultured without fibroblasts	39,02 ± 4,79	20,27 ± 3,55	16 ± 1,63	0
Cultured with fibroblasts	39,51 ± 2,11	44,47 ± 0,33	45,07 ± 0,75	45,6 ± 1,21
Group B				
Without fibroblasts	44,55 ± 3,54	26,47 ± 0,88	17,89 ± 0,56	0
With fibroblasts	50,13 ± 1,16	45,67 ± 0,67	47,33 ± 0,96	46,89 ± 0,68
Group C				
Without fibroblasts	0	4,68 ± 0,54	3,39 ± 0,2	0
With fibroblasts	0	0	0	0
Group D				
Without fibroblasts	16,42 ± 4,41	48,58 ± 4,17	62,73 ± 2,38	100
With fibroblasts	10,35 ± 0,95	9,86 ± 0,39	6,97 ± 0,37	7,51 ± 0,54

Comparison of the percentage of cells likely to have cilia (group A), cells being similar to basal cells (group B), cells being strongly PAS positive (group C), cells without affiliation (group D), with standard deviation

For respiratory epithelium grown in co-culture, the fracture of ciliated cells slightly increases from day 7 to day 14, persisting at a level of 44–45 % until day 33. Besides, the percentage of basal cells decreases slightly from day 7 to day 14, persisting at a stable level at 45–47 %. This suggests a differentiation of basal cells to ciliated cells between day 7 and day 14. Afterwards, the proportion between differentiated cells does not change. The occurrence of dedifferentiated cells shows a slight decrease until 21 days of culture to 6 %. No strongly PAS positive cells are found.

Summarized, Fig. 3 shows the dedifferentiation of respiratory epithelium grown without co-culture over time. Respiratory epithelium grown in co-culture stays differentiated into basal and ciliated cells until day 33.

### Immunohistochemical staining

**Fig. 4:**
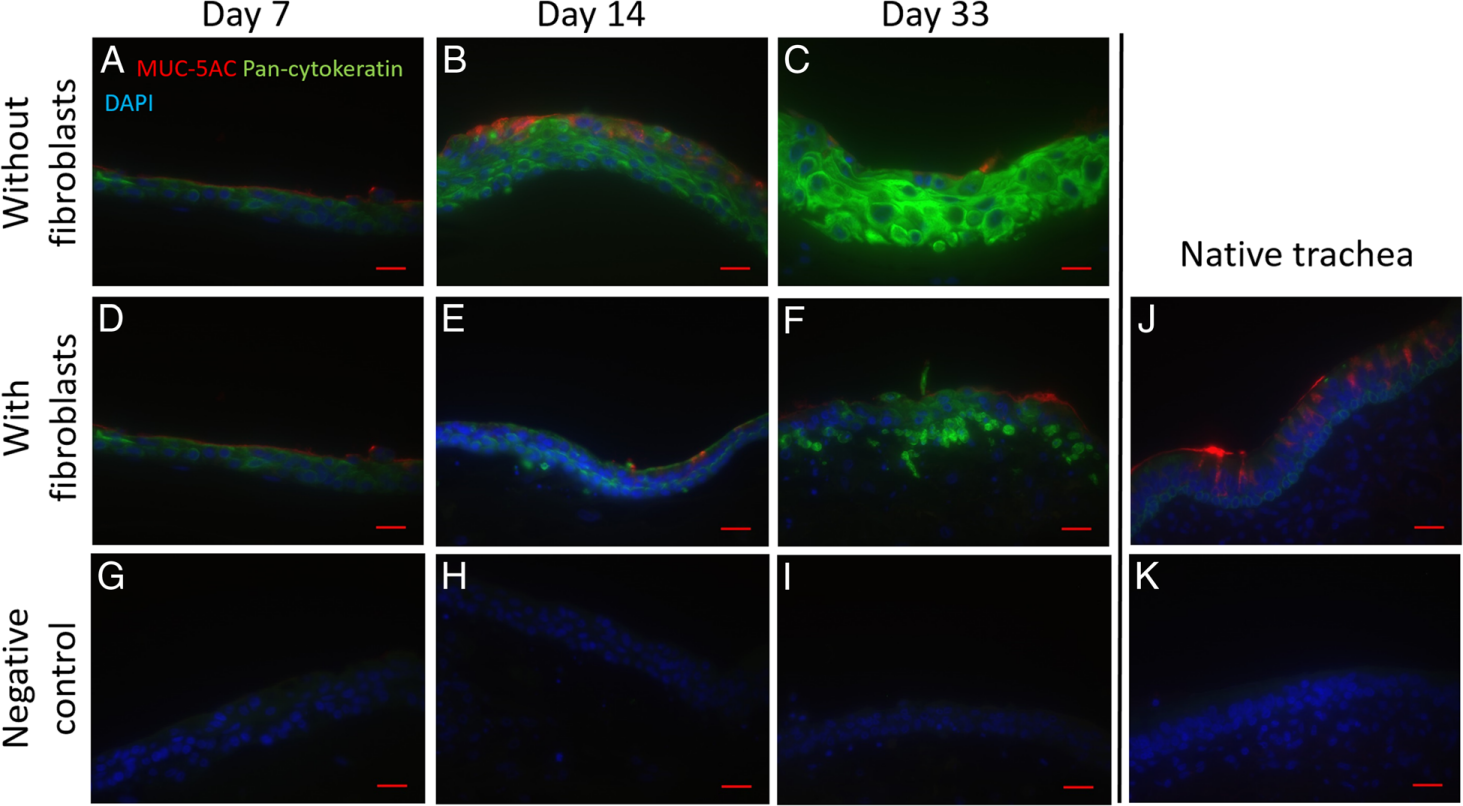
Immunohistochemical staining, pan-cytokeratin and MUC5AC. **a**-**c** Immunohistochemical staining against pan-cytokeratin and MUC5AC of respiratory epithelial cells seeded onto pure fibrin gel after 7, 14, 33 days of ALI-culture, respectively. Respiratory epithelial cells seeded onto pure fibrin gel showed a uniformly high expression of pan-cytokeratin in all cell layers. Some strongly MUC5AC positive cells are found at day 14. **d**-**f** Immunohistochemical staining against pan-cytokeratin and MUC5AC of respiratory epithelial cells seeded onto fibrin gel with embedded fibroblasts after 7, 14, 33 days of ALI-culture. Cells showed a similar pan-cytokeratin expression pattern to native trachea (**i**), with basal cells having a higher signal than apical cells and high signaling in the perinuclear area. Cells at all points of time carry a layer of MUC5AC positive mucus. **g**-**i**: Negative control of immunohistochemical staining against pan-cytokeratin and MUC5AC of respiratory epithelial cells seeded onto fibrin gel with embedded fibroblasts and respiratory epithelial cells seeded onto pure fibrin gel. **j** Native tracheal epithelium stained against pan-cytokeratin and MUC5AC, basal cells show a higher signal level of pan-cytokeratin than apical cells, the highest signaling occurs in the perinuclear area. **k** Negative control of immunohistochemical staining against pan-cytokeratin of native trachea. Scale: 20 μm

Immunohistochemical staining against pan-cytokeratin and MUC5AC reveals enhanced epithelial differentiation for respiratory epithelial cells cultured on the matrix containing fibroblasts. In native trachea, basal cells show a high signal level, whereas apical cells show a weak signal, and the highest signaling occurs in the perinuclear area. Respiratory epithelial cells seeded onto pure fibrin gel showed a uniformly high expression of pan-cytokeratin in all cell layers. Epithelial cells grown on fibrin gel with embedded fibroblasts showed a similar pan-cytokeratin expression to native trachea, with basal cells having a higher signal than apical cells. The expression of pan-cytokeratin developed more similarity to native tissue over time. After 14 days the pan-cytokeratin expression was akin to native tissue, having the same basoapical distribution, but varied in a general higher expression level (see Fig. 4).

MUC5AC staining shows occurrence of MUC5AC in all samples. There is no occurrence of cells with typical goblet cell morphology in both groups at any time. Cells grown on pure fibrin gel contain some strongly MUC5AC positive cells which matches the results of the quantification of cell differentiation. Cells grown on fibrin gel with embedded fibroblasts show a layer of MUC5AC positive mucus at day 7 and day 33 similar to native tissue, while lacking MUC5AC positive cells. The occurrence of MUC5AC proves that both groups consist of respiratory epithelial cells in diverging differentiation conditions.

**Fig. 5:**
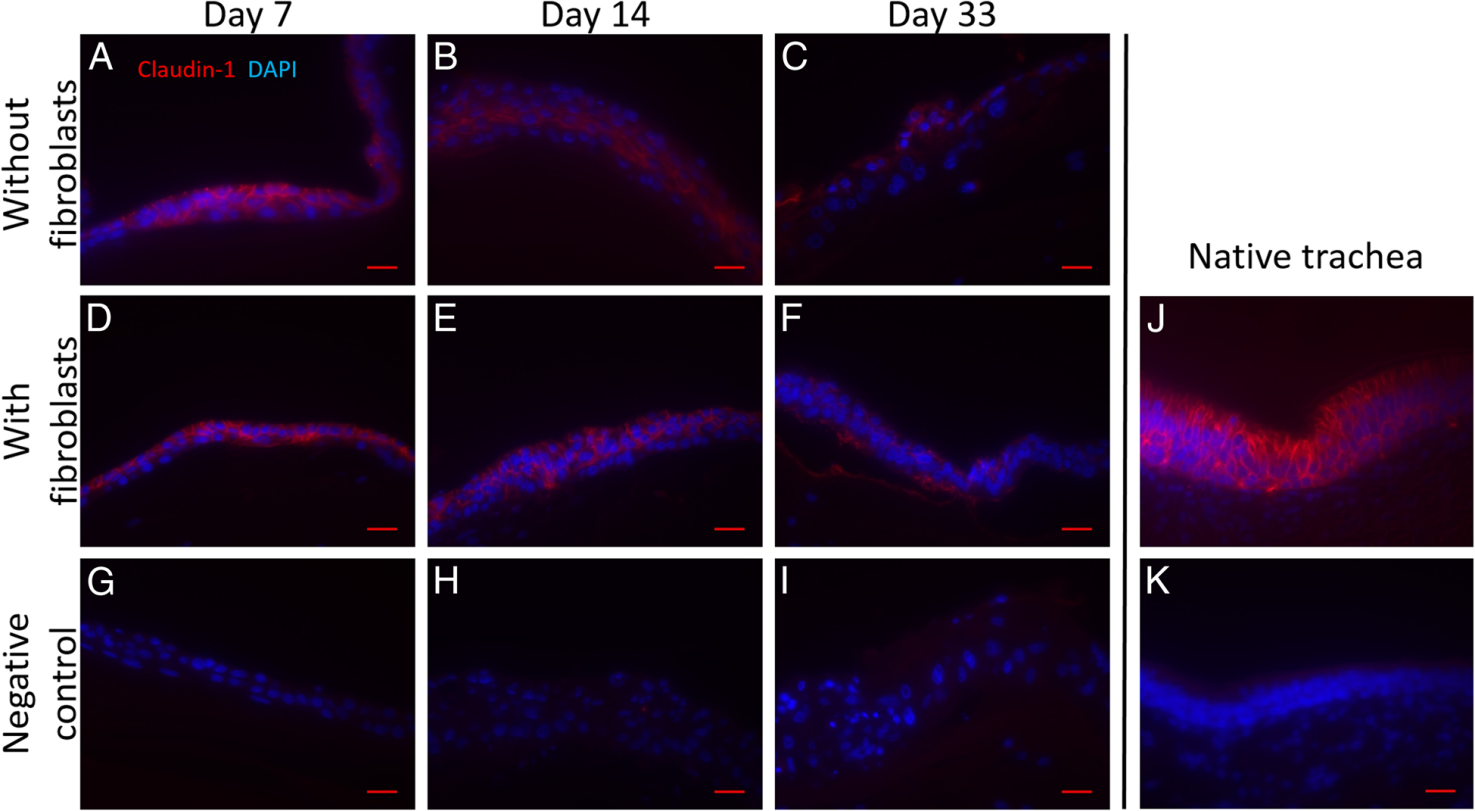
Immunohistochemical staining, claudin-1. **a**-**c** Immunohistochemical staining against claudin-1 of respiratory epithelial cells seeded onto pure fibrin gel after 7, 14, 33 days of ALI-culture, cells show a decreasing signal over time. **d**-**f** Immunohistochemical staining against claudin-1 of respiratory epithelial cells seeded onto fibrin gel with embedded fibroblasts after 7, 14, 33 days of ALI- culture. Cells stay positive until day 33 with an almost stable signal. A deposit of claudin-1 is seen to the fibrin gel at day 33. **g**-**i** Negative control of immunohistochemical staining against claudin-1 of respiratory epithelial cells seeded onto fibrin gel with embedded fibroblasts and respiratory epithelial cells seeded onto pure fibrin gel. **j** Native tracheal epithelium stained against claudin-1, a high signal level is seen at all cell boundaries. **k** Negative control of immunohistochemical staining against claudin-1 of native trachea. Scale: 20 μm

Figure 5 shows the occurrence of claudin-1, which is a marker of tight junctions and, therefore, an important marker of the epectrical barrier function of the epithelium. After 7 days there is no difference between cells cultured without fibroblasts and cells in co culture between the distribution and signal of claudin-1, the signal level is slightly lower compared to native trachea. In cells grown on pure fibrin gel the signal level decreases over time leading to a poor expression level after 33 days. Cells grown on fibrin gel with embedded fibroblasts show a stable the signal level over time, being commonly lower than in native trachea. After 33 days a deposition of claudin-1 onto the fibrin gel is found. This proves that seeded cells are epithelial cells. Cells grown on pure fibrin gel lose their barrier function over time.

## Discussion

We hypothesized that co-culture with fibroblasts might improve the differentiation of respiratory epithelium in a three-dimensional tissue model based on fibroblasts embedded into fibrin gel. For evaluation of the differentiation, we used histological staining - PAS - and immunohistochemical staining against pan-cytokeratin. We found that differentiation and organization of respiratory epithelium cultured atop fibroblasts increases over a period of 33 days. In contrast, respiratory epithelial cells grown on pure fibrin gel dedifferentiated and showed hyperproliferation after 14 days of culture, resulting in a mainly dedifferentiated and unorganized epithelium at day 33. The morphology of respiratory epithelial cells cultured without fibroblasts is comparable to squamous epithelium. This indicates that fibroblasts not only support differentiation of respiratory epithelial cells, but also inhibit dedifferentiation of respiratory epithelial cells. We observed a ciliated cell layer on respiratory epithelial cells cultured atop fibroblasts. Co-cultured epithelium developed more ciliated cells than epithelium cultured atop pure fibrin gel. Hence co-culture with fibroblasts seems to support development of ciliated cells and might therefore facilitate mucociliary clearance.

The epithelialization of tracheal transplants is important for fast integration of an intact epithelial barrier with proper ion and water homoeostasis and mucociliary clearance to avoid fibroblastic overgrowth, inflammatory responses and infections [10]. We concluded that a proper differentiation is essential for the especially critical early time after implantation of tracheal implants.

The establishment of a well differentiated respiratory epithelium is not only an aim of tissue engineering of implants. Bezhad et al. found fibroblasts are involved into a remodeling process of the epithelial mesenchymal trophic unit in COPD. Reduction of epithelial-fibroblast-contact was seen in mild and severe COPD [11]. Likewise in Asthma interactions between epithelium and underlying mesenchyme seem to interact in maintain chronic inflammation of the airways [12]. Therefore, establishing an in vitro model for COPD and asthma with fibroblasts is crucial for understanding underlying pathomechanisms and observe the mechanisms of new pharmaceutical options for clinical application in vitro.

To investigate mechanisms *in vitro*, different co-culturing systems have been established to simulate airway remodeling in tracheobronchial epithelium. Pageau et al. described a system which consists of a type one collagen matrix, normal human lung fibroblasts, and a surface epithelium of normal bronchial epithelial cells [13]. Choe et al. built a system in which they suspended fibroblasts in a collagen matrix and put differentiated respiratory epithelium on top for investigation of asthma [8].

In terms of tissue engineering of a tracheal implant, co-culture systems seem to be useful to induce differentiation of respiratory epithelial cells. Main aim in these systems is to promote differentiation and migration of respiratory epithelial cells and prevent dedifferentiation. One approach is suspending fibroblasts in a collagen gel [14]. Another is to co-culture fibroblasts on different sides of an amnion membrane [7].

Mechanisms of differentiation of epithelial cells are not fully understood. Goto et al. found that when using preconditioned media instead of fibroblasts themselves respiratory epithelial showed the same improvement of differentiation compared to co-culturing with tracheal fibroblasts [7]. Thus, endocrine or paracrine factors must play a major role in epithelial differentiation. Many factors influencing epithelial regeneration have been described. Important factors known to improve differentiation of respiratory epithelium are epidermal growth factor (EGF), retinoic acid, hepatocyte growth factor and transforming growth factor (TGF) β [15–19]. Gray’s medium already contains EGF and retinoic acid in a high concentration of 50 μM. Thus fibroblasts must secrete other factors, such as hepatocyte growth factor for differentiation of respiratory epithelial cells. However, this needs further investigation.

It is well understood that respiratory epithelial cells need scaffolds mimicking the microenvironment to support differentiation, migration and polarity [10]. Extracellular matrix (ECM) and the re-modeling of ECM by metalloproteinase by respiratory epithelial cells and fibroblasts seem to enhance cell migration and proliferation as well as differentiation of migrating cells [20]. Other studies showed that fibroblast in co-culture promote secretion of ECM and the buildup of a basal membrane with laminin and type IV collagen at the bottom of respiratory epithelial cells [14].

Collagen is long known to support respiratory epithelial differentiation [21]. Many scaffolds used in respiratory tissue engineering have collagen as major component [22–24]. Collagen seems to be especially important for the ciliated cell differentiation [21] and influences the profile of secretions of respiratory epithelium [25]. For tissue engineered implants, it has several disadvantages. For example it cannot be produced in an autologous way as easy as fibrin gel and has weak mechanical properties [5].

We hypothesize that the interaction of paracrine, autocrine and indirect interaction via ECM production of the fibroblast leads to the promotion of the mucociliary differentiation of respiratory epithelium. Further investigation on which factors influence the epithelium and the fibroblasts in our model is required.

On the account of our and the studies stated before, we think that fibroblasts are an essential component of a tissue engineered trachea for promoting and keeping up differentiation of the respiratory epithelium over time and to support epithelial migration to sustain mucociliary clearance. This could resemble a step towards a tissue engineered tracheal implant being employed in a clinical setting.

In contrast to other groups, we employed fibrin gel for embedding fibroblasts since it has many advantages in terms of tissue engineering. Complex geometries can be produced by injection molding technique, autologous production from patient’s blood and its effective cell seeding process through embedding cells into fibrin gel during the molding process made fibrin gel an often used scaffold for tissue engineering. Also, fibrin accelerates wound healing, [6] has adhesive properties and provides intrinsic growth factors to promote epithelial cell and fibroblast migration, proliferation and angiogenesis [26]. Different applications are possible because of its potential as a drug delivery system [27]. In another approach fibrin gel has been applied as a glue for transferring fibrous capsules containing respiratory epithelium for re-epithelialization of a trachea [28]. We already proved the suitability of fibrin gel as a scaffold for respiratory tissue engineering in a previous study [5]. This study promotes the advantage of fibrin gel with its positive effect on the growth of fibroblasts and thus positively influences epithelial differentiation.

## Conclusion

In this study, we evaluated the influence of respiratory epithelial cells co-cultured with fibroblasts in a fibrin gel. After four weeks of *in vitro* culture, morphology of the co-cultured respiratory epithelium resembled native tracheal epithelium. Immunohistochemistry showed that respiratory epithelium with fibroblasts had an increasing similarity of pan-cytokeratin expression compared to native trachea. Cells cultured without fibroblasts differed in pan-cytokeratin expression from native trachea and did not show any improvement of differentiation. Thus, our study indicates that adding fibroblasts to fibrin gel positively influences the differentiation of respiratory epithelium.
